# Multiomics and blood-based biomarkers of electroconvulsive therapy in severe and treatment-resistant depression: study protocol of the DetECT study

**DOI:** 10.1007/s00406-023-01647-1

**Published:** 2023-08-30

**Authors:** Iven-Alex von Mücke-Heim, Julius C. Pape, Norma C. Grandi, Angelika Erhardt, Jan M. Deussing, Elisabeth B. Binder

**Affiliations:** 1 Department Genes and Environment, Max Planck Institute of Psychiatry, Kraepelinstraße 2-10, 80804 Munich, Germany; 2 Research Group Molecular Neurogenetics, Max Planck Institute of Psychiatry, Kraepelinstraße 2-10, 80804 Munich, Germany; 3 Department of Psychiatry, Clinical Anxiety Research, University of Würzburg, Josef-Schneider-Straße 2, 97080 Würzburg, Germany

**Keywords:** Treatment resistance, Major depressive disorder, Multiomics, Electroconvulsive therapy, Biomarkers

## Abstract

Electroconvulsive therapy (ECT) is commonly used to treat treatment-resistant depression (TRD). However, our knowledge of the ECT-induced molecular mechanisms causing clinical improvement is limited. To address this issue, we developed the single-center, prospective observational DetECT study (“Multimodal Biomarkers of ECT in TRD”; registered 18/07/2022, www.clinicalTrials.gov, NCT05463562). Its objective is to identify molecular, psychological, socioeconomic, and clinical biomarkers of ECT response in TRD. We aim to recruit *n* = 134 patients in 3 years. Over the course of 12 biweekly ECT sessions (± 7 weeks), participant blood is collected before and 1 h after the first and seventh ECT and within 1 week after the twelfth session. In pilot subjects (first *n* = 10), additional blood draws are performed 3 and 6 h after the first ECT session to determine the optimal post-ECT blood draw interval. In blood samples, multiomic analyses are performed focusing on genotyping, epigenetics, RNA sequencing, neuron-derived exosomes, purines, and immunometabolics. To determine clinical response and side effects, participants are asked weekly to complete four standardized self-rating questionnaires on depressive and somatic symptoms. Additionally, clinician ratings are obtained three times (weeks 1, 4, and 7) within structured clinical interviews. Medical and sociodemographic data are extracted from patient records. The multimodal data collected are used to perform the conventional statistics as well as mixed linear modeling to identify clusters that link biobehavioural measures to ECT response. The DetECT study can provide important insight into the complex mechanisms of ECT in TRD and a step toward biologically informed and data-driven-based ECT biomarkers.

## Introduction

Major depressive disorder (MDD) is among the most prevalent disorders worldwide [1, 2]. Its 12-month prevalence is ~ 11% and women are affected twice as often as men [3, 4]. It is associated with high personal and socioeconomic costs, also through as well as an elevated morbidity compared to the general public [5–7]. In addition, patients with MDD experience higher cardiovascular and metabolic comorbidities, which contribute to the observed excess mortality [6, 7]. MDD is an episodic disorder and recurrence affects 40–50% [8]. A chronic course of disease (> 24 months) is found in about 25% of MDD subjects [9]. While a number of effective treatments exist, a high proportion of MDD patients do not sufficiently respond to current therapies. Treatment resistance (treatment-resistant depression, TRD) affects about 30% of even patients and is accompanied by higher rates of psychiatric and somatic morbidity as well as mortality [10–12]. TRD definitions are somewhat heterogeneous [13–15], yet the most common one is a lack of clinically meaningful improvement (non-response) following two antidepressant drugs administered in an appropriate dosage and duration [13, 16–18]. This definition is shared by the U.S. Food and Drug Administration (FDA) [19] as well as the European Medicines Agency [20] and the recent German federal depression treatment guidelines [21]. TRD is an important medical and societal issue [22], especially taking the globally rising incidence into account [23–25]. One in three TRD patients attempts suicide at least once in their lifetime, which is twice as high as in treatment-responsive depression [26]. TRD patients use health services more frequently and generate high healthcare costs, both at the absolute level as well as proportionally to the overall costs related to depression [27–29].

Beyond conventional pharmaco- and psychotherapy, neurostimulation techniques such as electroconvulsive therapy (ECT) are widely available, safe, and powerful treatment options in psychiatry [30–33]. About 80% of all ECTs are performed in patients with MDD [34, 35]. Though ECT should always be preceded by an individual risk–benefit assessment [36], studies and meta-analyses overall report markedly favorable response rates as high as 79% and remission in up to 70% of cases [37–39]. In severe and psychotic depressive episodes, Petrides et al. have even reported remission in 95% of their participants [40]. ECT is also a highly effective treatment option for TRD [37], with an efficacy comparable to the general response rates seen in treatment-responsive depression [41]. Studies and meta-analyses indicate an ECT-related response in 56–85% and a remission in about 48% of patients with prior medication failure or TRD, respectively [27, 41–44]. This response rate in TRD is similar to the response rates reported for antidepressant drugs and psychotherapy overall in non TRD patients of about 50–60% [45–51]. It is thus not surprising that the mean effect size of ECT (d ≈ 0.9–1.3) is higher than the one of antidepressants (*d* ≈ 0.37) or psychotherapy (*d* ≈ 0.75) [52–58]. ECT-induced remission is commonly achieved early during treatment (~ 3 weeks after ECT start) [27, 59]. ECT is also known to significantly reduce the risk of suicide [33, 60–62]. Due to the high recurrence rates of up to 81% after acute-phase ECT, maintenance sessions are recommended and performed in gradually decreasing frequency to preserve the initial therapy success [36, 39, 63–68]. During an ECT series, sub-/acute side effects, such as headaches, muscle soreness, nausea, transient hypertension, anterograde and retrograde amnesia (especially ± 3 days around the ECT), and postictal confusion, rarely a delirium, can occur due to the applied current and induced seizure apart from general anesthesia risks [69, 70]. These adverse effects are reversible and short-lived in the majority of cases (< 1 h post-ECT) [69]. The frequency of cognitive or memory side effects is associated with patient and disease characteristics as well as the ECT stimulation paradigm and intensity (e.g., strong, bitemporal stimulation over a long period of time: increased memory disturbance) [70–73]. On average, however, cognitive performance tends to remain the same or improve after ECT treatment [71, 74, 75]. Both magnetic resonance imaging and autopsy studies as well as animal experiments have not demonstrated any organic brain damage due to ECT [76–80]. The balance between strong clinical effects and a tolerable side effect profile makes ECT the gold standard of stimulation methods, provided appropriate patient selection [55, 75, 81, 82].

The therapeutic effect of ECT is thought to come from a combination of short- and long-term effects of the induced epileptic seizures [83]. There is evidence from both preclinical as well as clinical studies on possible molecular/biological mechanisms of action, but not always consistent across studies. The most important findings and hypotheses, however, can be summarized as follows: increase in neurotrophic factors such as brain-derived neurotrophic factors (BDNF) and enhanced neuroplasticity including the formation of new synapses, modulation of neurotransmitter metabolism and subsequent alterations in specific neurotransmission accompanied by modification of epigenetic processes and gene expression changes. Alterations in immune cell composition and cytokine levels and regulation and normalization of hypothalamic–pituitary–adrenal axis dysregulation have been reported. From neuroimaging studies, changes in gray and white matter volumes and normalization of altered functional connectivity are reported [27, 69, 83–85]. To date, however, it is not clear which of these biomolecular processes are relevant for the therapeutic success of ECT [86, 87]. Accordingly, no reliable and clinically applicable biomarkers for ECT are available for MDD and TRD [82, 88–92]. Due to this lack of objective biological measures, clinical decision on ECT is still solely informed by consensus-based treatment guidelines [36, 93, 94]. Similarly, prediction of ECT success is loosely based on a few clinical parameters like episode duration and severity [95, 96], failed prior pharmacotherapy [42, 43], age [97, 98], psychotic features [40], and early symptom improvement [99]. In order to improve clinical decision making and identify MDD patients with an optimal ECT risk–benefit profile, reliable biomarkers and multimodal prognosis models are urgently needed [40, 75]. Although a handful of studies have suggested potential biomarkers [100–113], so far none has proven valid or reliable enough for routine application in day-to-day care.

We have initiated the DetECT study to improve our understanding of the molecular mechanisms contributing to response to ECT in TRD and to discover novel biomarkers for clinical application. Our study relies on a multilevel data structure and multiomics approach including novel potential biomarkers such as: (i) neural cell-derived exosomes, as they hold the potential to serve as peripheral biomarkers mirroring ECT-induced molecular changes in the brain [114–117]; and (ii) purinergic signaling dynamics, potentially modulating immune mechanisms linked to treatment resistance and ECT response [118–121]. Our multidimensional data collection is described below and acknowledges the inherent complexity of MDD and the subsequent biobehavioral changes and patterns evoked by ECT. Ultimately, future biomarkers will likely be a cluster of molecular, behavioral, and clinical measures rather than one single parameter [70, 99].

## Methods

**Fig. 1:**
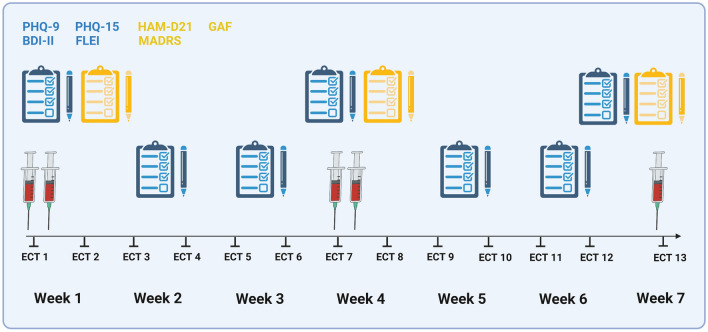
Overview of serial blood draws and psychometry in the DetECT study. In the study’s main phase, blood draws are performed before and after the first and seventh as well as within 1 week after the twelfth ECT session. To estimate clinical response and side effects over time, participants are asked weekly to complete four standardized self-rating questionnaires on depressive and somatic symptoms (Patient Health Questionnaires 9 and 15, Questionnaire on Mental Capacity, Beck Depression Inventory II). Foreign ratings are obtained thrice by a standardized clinical interview (Montgomery-Åsberg Depression Rating Scale, Hamilton Rating Scale for Depression, Global Assessment of Functioning) (created with www.Biorender.com)

The DetECT study was initiated at the Max Planck Institute of Psychiatry’s (MPIP) research clinic in 2022. All study documents have been approved by the local ethics committee of the Ludwig-Maximilians-University Munich (Project registration number: 21–1087); see also https://www.psych.mpg.de/detect. The study is enrolled with the U.S. National Institute of Health (NIH) clinical study register www.ClinicalTrials.gov (Study ID: NCT05463562, registration date 18/07/2022). The project is conducted in strict accordance with all relevant national and international regulations including the Declaration of Helsinki. A detailed schematic of the study design can be found in Fig. 1.

## Study design and population

The DetECT project has a prospective and exploratory design. Recruitment is planned to last 3 years. The study is conducted exclusively in adult (≥ 18 years) and legally competent severely depressed in-patients receiving treatment at the MPIP’s research clinic in the form of ECT. The study design is purely observational. Therefore, both the diagnostic and treatment process including the mode of ECT administration as well as pharmacotherapy is determined solely by the attending psychiatrist in joint decision with the patient based on the overall clinical situation in accordance with the German depression treatment guidelines [21]. Diagnosis is made by a trained psychiatrist in compliance with the depression criteria of the International Classification of Diseases 10th revision (ICD-10). Patients included in the study suffer from TRD that is defined in accordance with the FDA and EMA guidelines, i.e., two or more failed antidepressant treatments in adequate dosing and duration. Depression severity is evaluated via standardized self- and clinician ratings, as described later in detail. Inclusion and exclusion criteria are listed in Table 1.

**Table 1:** DetECT eligibility criteria

Inclusion criteria
Age ≥ 18 years, legally competent, and desire to participate
Diagnosis of a depressive episode according to the ICD-10
Severity is confirmed using self- and foreign-rating psychometry
Treatment resistance (i.e. ≥ 2 failed antidepressant treatments)
Clinical indication and upcoming electroconvulsive therapy
Signed electroconvulsive therapy informed consent form
Signed informed consent documents for the DetECT study
Signed consent and participation in MPIP’s biobanking project
Proficient German language and communication skills
Exclusion Criteria
Age < 18 years (minor) or no desire to participate
Legal supervision or acute legal incompetence
Pregnancy and/or breastfeeding
Pervasive developmental disorders and/or intellectual disability
Acute substance abuse (e.g., alcohol, prescription, or illicit drugs)
Severe neurological disease (e.g., severe organic brain damage)
Acute or serious general illness (e.g., relevant anemia requiring transfusion)

In study participants, serial venous blood draws (prior fasting: ≥ 6 h) are performed before and after the first and seventh as well as after the twelfth ECT session. A detailed schematic of the study design is given in Fig. 1. Pre-ECT blood draws with a total volume of 36 ml include collection of ethylenediamine tetra acetic acid (EDTA) (Sarstedt, S-Monovette® K3 EDTA) and serum (Sarstedt, S-Monovette® Serum Gel) tubes as well as PaxGene Blood RNA Tubes (Qiagen) for the extraction and analysis of DNA and epigenetic measures (e.g., methylation patterns), RNA including messenger and micro-RNA (mRNA and miRNA, respectively), neuron-derived exosomes, purines, hormones, proteins, electrolytes, metabolic parameters, inflammatory markers, and peripheral blood mononuclear cells (PBMCs). Post-ECT blood draws include only EDTA and serum tubes (Sarstedt) for total of 10 ml. In a subset of *n* = 10 participants, which are recruited at the very beginning of the study’s field phase (DetECT pilot phase), the ECT blood collection after the first ECT consists of three time points (1 h, 3 h, and 6 h post). The rationale for this schedule is to characterize the temporal post-ECT dynamics. This would also allow to discriminate between an immediate ECT-induced release of brain-derived molecules and cells through the blood–brain barrier (fast pathway) or a delayed release via cerebrospinal fluid drainage (slow pathway). To achieve this, the pilot phase materials and data are analyzed with regards to the different multiomics read-outs to determine the optimal post-ECT time point for the study main phase. To reduce batch effects, samples are stored in the MPIP’s biobanking unit in freezers for later analyses with larger sample sizes.

In addition to the serial blood draws, participants are asked to answer the following self-rating questionnaires concerning their depressive and somatic symptoms on a weekly basis: Patient Health Questionnaires 9 and 15 (PHQ-9/-15) [122–125], the Questionnaire on Mental Capacity (FLei) [126], and the Beck Depression Inventory (BDI-II) [127–129]. The reference-interval of the self-rating questionnaires was customized from two (PHQ-9, BDI-II) and four (PHQ-15) weeks as well as six month (FLei) to the week prior to match the DetECT study design. Moreover, three clinician-rating instruments are assessed via a clinical interview in week 1, 4, and 7 of the study period: the Montgomery-Åsberg Depression Rating Scale (MADRS) [130, 131], the Hamilton Rating Scale for Depression with 21 items (HAM-D21) [132–134], and the DSM-IV derived Global Assessment of Functioning (GAF) [135, 136]. In addition to study related procedures, metabolic parameters (i.e., body mass index (BMI) or blood pressure (BP)) as well as medical information (e.g., patient and family anamnesis, past and recent medication including concomitant psychopharmacological treatment pre and during ECT allergies, drug use, psychiatric and somatic diagnoses) and sociodemographic data are extracted from patient records. See Fig. 1 for further detail.

## Screening and recruitment

All patients who receive inpatient psychiatric treatment including a pending ECT for a confirmed TRD are informed about the DetECT study and the possibility of participating. This is done by the treating/responsible psychiatrist or another treatment team member. Rarely, first contact with the study team is made through an interested patient directly. If patients express an interest in participating, after receiving first and general information about the DetECT study, their eligibility and contact information is passed onto the MPIP Study Center. Study participation is completely voluntary. After obtaining the signed informed consent form, all study-related procedures are commenced.

## Study timeline

**Fig. 2:**
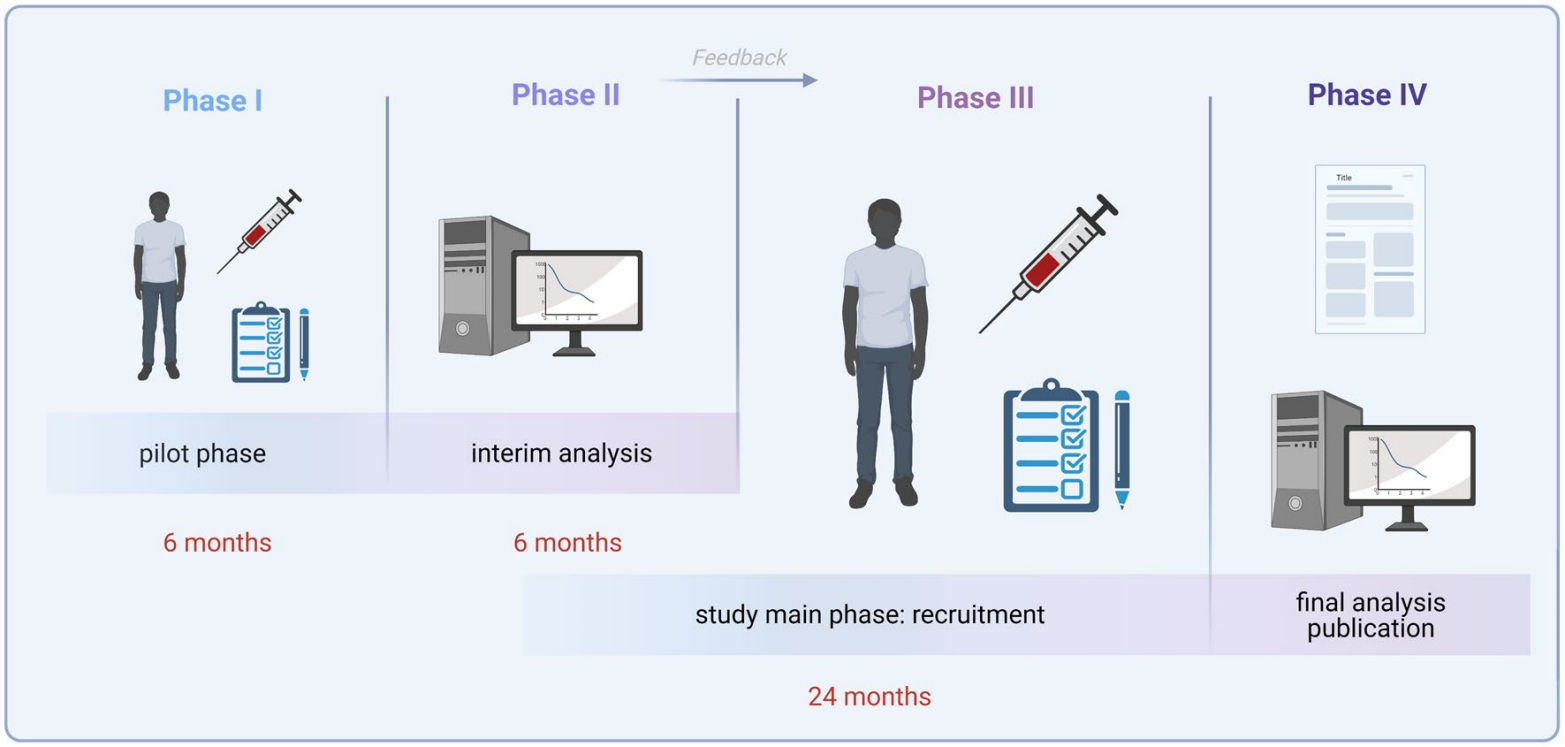
Schematic DetECT study timeline. The study period can be divided in four phases: (I) recruitment of the *n* = 10 pilot participants; (II) analyses of pilot materials and data. Primarily highly dynamic parameters (e.g., RNA expression, neuron-derived exosomes, immune cells, cytokines, and purines) are measured and validated. (III) Study main phase aiming to recruit a total of *n* = 124 participants over the course of 2 years. (IV) Finally, data are analyzed and published with a focus on the potential of the novel insights and biomarkers for clinical application (created with www.Biorender.com)

The study period can be divided into four distinct consecutive work phases (see Fig. 2). Phase one commenced in September 2022 and will recruit minimum of the n = 10 pilot participants. It is estimated to last half a year. After completion, pilot materials and data are analyzed (Phase two). Here, measurements of highly dynamic parameters (e.g., RNA expression, neuron-derived exosome analyses, and immunological measures including cytokines and purines) are carried out and validated methodically. We expect this to yield meaningful and novel information about the post-ECT temporal dynamics of different biological read-outs. The focus of the laboratory and statistical analyses is at which time (i.e., either after 1 h, 3 h, or 6 h) potential biomarkers show the largest deflection from baseline. The timeframe for these analyses is expected to be half a year. Based on the biomarker dynamics of the pilot samples, the post-ECT blood collection interval for the DetECT main phase (Phase three) will be determined. In the main phase of the study, a total of *n* = 124 participants will be recruited over the course of 2 years. Finally (Phase four), the data obtained are analyzed and published. Here, the focus is not only on the creation of new scientific knowledge, but the clinical implementation of the identified biomarkers.

## Longitudinal psychometric measures

To simultaneously measure depressive, neurocognitive, and somatic symptoms longitudinally in DetECT participants, we have selected four self-rating and three clinician-rating instruments. Since DetECT patients suffer from severe TRD and are thus heavily affected, psychometry was tailored to minimize participant efforts, while maximizing read-outs and item validation options post hoc. Only German questionnaire versions were used (see [126, 131, 137–140]).

Self-rating questionnaires: The PHQ-9 and PHQ-15 are both subscales of the patient health questionnaire (PHQ), which have proven effective for monitoring depression and somatic symptom burden [141]. The PHQ-9 is a self-administered depression screening, which contains a dimensional answer category (0 = not at all; 3 = nearly every day) for the nine depression criteria derived of the DSM-IV [123, 142]. The BDI-II is, similar to the PHQ-9, a multiple-choice self-report questionnaire with high validity and reliability [127–129, 143]. It is composed of 21 items, which have values of 0 to 3, to assess the presence as well as severity of depression [127]. The PHQ-15, on the other hand, is a self-rating questionnaire to assess somatic symptom load via 15 questions (0 = not bothered at all; 2 = bothered a lot). Summary scores are used as indicators of somatic symptom burden [125]. The FLei is a comparatively novel measure of neurocognitive function. By use of 35 questions with answers ranging from 0 = never to 4 = very frequently, the following domains are assessed: (I) attention, (II) memory, (III) executive functions, (IV) control scale [126]. Based on the FLei domain and sum scores, a longitudinal neurocognitive profile can be computed.

Clinician-rating questionnaires: Both, the MADRS and HAM-D21 are obtained by trained study personnel in a clinical interview. The MADRS (10-question instrument) and the HAM-D21 (21 items) both assess depression presence and severity [130–134]. The GAF is a functionality and severity score for psychiatric disorders ranging from 0 (= maximum symptom severity and load, extreme sociofunctional impairment) to 100 (= no symptoms or impairment). The index accounts for overall psychiatric symptom severity and sociofunctional impairment [135, 136].

## Sample analytics: blood-based multiomics and biomarker screening

Using the collected and frozen within-subject blood samples, we aim to perform multiomics and deep phenotyping. To reduce batch effects and overall experimental variability, all laboratory measurements for a certain measure are performed together.

### Genotyping and epigenetic analyses

Using DNA isolated with a conventional extraction kit from EDTA blood, genotyping and epigenetic profiling will be performed using array-based approaches. Quality is ensured due to our in-house standard operating procedure.

### mRNA sequencing

Following mRNA extraction from thawed EDTA blood stored in PaxGene Blood RNA Tubes (Qiagen), we will use commercially available Library Prep Kits to generate sequencing libraries. Libraries will be multiplexed and sequenced on an Illumina® platform.

### Neuron-derived exosomes and cargo analysis

Neuronal exosome isolation will be performed based on the protocol by Saeedi and Nagy et al. 2021 [144]. In brief, exosomes extracted from EDTA-plasma will be enriched using neuronal markers (e.g., synaptosomal-associated protein of 25 kDa, SNAP-25). Subsequently, exosome cargo will be analyzed focusing on miRNAs.

### Cytokine and immune metabolite quantification

For cytokine quantifications, we plan to use custom-designed high-sensitivity immunomagnetic assays. Cytokine panels will be designed to reflect both pro- and anti-inflammatory states, in parts based on findings from large meta-analyses on cytokine changes in MDD [145]. Additionally, we will conduct ELISAs targeting other immunogenic metabolites like kynurenine and quinolinic acid [146].

### Purine measurements

Total purine levels (mM) are measured immediately after venous blood collection in 40 µl of whole blood using high-sensitivity electrochemical sensors (Zimmerman & Peacock). Serum uric acid levels from routine blood draws at admission and comorbid gout and/or anti-gout medication is extracted from patient records and used as a covariable.

### Immune cell phenotyping

To characterize immune profiles beyond cytokine clusters, we intend to perform both flow cytometry and fluorescence-activated cell sorting (FACS) using the acquired PBMCs. Based on changes reported in prior ECT studies [84, 147–152], we will use surface and intracellular markers to characterize immune cell subpopulations.

### Metabolic and endocrine profiling

In addition to humoral and cellular immune profiling, we aim to characterize metabolic and endocrine states using established clinical and blood markers (e.g., BMI, BP, serum lipids, leptin and ghrelin, PBMC membrane proteins like CD220 or GLUT1, corticotropin-releasing hormone, and cortisol). This approach aligns with the growing evidence linking immunometabolic and neuroendocrine dysregulation to MDD [153–158].

## Study hypotheses and objectives

The primary hypothesis of the DetECT study is that there exists a joint biological function and clinically meaningful connection between changes in gene expression and regulation, immunometabolic conditions, purinergic signaling, and CNS-derived exosome release in TRD patients receiving ECT. The secondary hypothesis is that these changes can be utilized to predict and monitor ECT outcome in TRD patients.

The main study objective is therefore to identify biological, psychological, socioeconomic, and clinical parameters (biomarkers) that provide detailed information about the general or individual factors of ECT success and the biobehavioral disease subgroups these changes are linked to. The DetECT data may also serve as a cornerstone for the development of a biologically founded, multimodal prognosis model for ECT use in TRD. As a subordinate objective, we hope to generate novel insight into the molecular effects of ECT in TRD using the generated omics data. Taken together, we believe that the DetECT results can aid precision medicine approaches for ECT application and to boost its social as well as clinical acceptance.

## Power calculation

In complex psychiatric illnesses, such as MDD, which arise from the reciprocal interaction of endogenous and exogenous influences (interaction: genes x environment) [159], the effect of single parameters is small. Only the interaction of many and qualitatively heterogeneous components leads to the disease. The best-known explanatory model is the diathesis-stress model, in which the interaction of individual vulnerability (biological and psychosocial) and external influences is acknowledged. Therefore, and due to the exploratory and multiomics approach of the DetECT study focusing on unstudied parameters, sample size calculation cannot simply be performed based on preexisting study results. Accordingly, we have based our power calculation on the clinical ECT efficacy [55, 56], as it is a composite indicator of the interplay of multiple ECT-induced biological mechanisms and their effect sizes, respectively. Using G*Power 3 Software comparing responders with non-responders assuming a moderate effect size of *d* = 0.5, a significance level *α* = 0.05, and a power of 1–*β* = 0.8, we computed a sample size of *n* = 134 [160].

## Prospective statistical analysis

The statistical analysis of the DetECT study mainly focuses on comparing ECT responders with non-responders (internal control group) within the sample. Clinical response indicated by overall and sub-domain changes in psychometry will serve as main dependent variables and analyses focus on within-subject changes over time. On the one hand, we aim to use variance and correlation analyses (e.g., repeated-measures ANOVA, Pearson’s r and correlation matrices), regression models (e.g., linear or logistic regression), and principal component analysis to first identify both significant main and interaction effects between biobehavioral parameters and treatment response. These analyses will be performed for all measures pre-, during, and post-treatment. Aside from using single parameters and the area under the curve as independent variables, we will employ index and score approaches (e.g., inflammatory indexing and Z scores) to allow combined analyses of functionally connected processes. Following these steps, we plan to employ linear mixed models (LMM) to examine if a single or parameter cluster is linked to remission, treatment response, failure, and side effects. As described above, we will also use single and combined as well as over time parameters, indices, or scores to perform LMM. Factors like the SES, comorbidities, smoking, concomitant pharmacological treatment (both general and psychiatry specific), and mode of ECT application (e.g., right-unilateral vs. left-anterior-right-temporal) will be used as covariates. Multiple comparisons correction will be applied. To further ensure that the identified associations are not only statistically significant, but also represent a relevant underlying biological mechanism, we will report both significance (p value) as well as effect size (e.g., Cohen’s d or comparable measures) in prospective DetECT publications, as recommended for instance by Goodman et al. 2019 [161].

## Conclusion and outlook

The DetECT study is among the first trials to conduct multiomics-based biomarker screening using longitudinal within-subject blood collection and psychometry combined with clinical information in TRD subjects receiving ECT. The expected multilevel data structure will aid the generation of novel knowledge on both disease and therapy response mechanisms on a clinical, cellular, and molecular level. These insights will then enable a targeted search for biomarkers associated with beneficial outcomes and biobehavioral depression subtypes. Ideally, these findings will also be a first step toward a clinically applicable tool to predict ECT efficacy in TRD patients. The latter would ultimately pave the way toward personalization and specification of ECT application.

## Data Availability

The data generated and analyzed contain clinical data that cannot be made publicly available due to privacy rights of participants. In principle, data may be made available given a legitimate and science-related request.
